# Associations between body composition and physical performance in Chinese male adults: a cross-sectional study

**DOI:** 10.3389/fpubh.2026.1876542

**Published:** 2026-06-16

**Authors:** Hong-Mei Chen, Meng-Yuan Shu, Xiu-Chang Zhang, Yong Yang, Li Shao, Xiao Zhang, Di Lu, Mingyi Ma, Yiqian Li, Guzalinur Yushan, Yunrui Liu, Lintao Wang, Chi Duan, Ru-Xue Yin, Wen Fang, Xiong Xiong, Ying-Hong Xiong, Li Liu, Jian Liang

**Affiliations:** 1 Department of Sports Science, Hengyang Normal University, Hengyang, China; 2 School of Physical Education, Kashi University, Kashi, China; 3 Department of Sports Medicine, Soonchunhyang University, Asan-si, Republic of Korea; 4 Experimental Teaching Demonstration Center of Food Safety and Nutrition, Xinjiang Institute of Technology, Aksu, China; 5 Aksu Institute of Apple, Xinjiang Institute of Technology, Aksu, China; 6 Hengyang Scientific Research Tutoring Station, Hengyang, China; 7 Furong School of Taiyuan Town, Hengyang, China; 8 Faculty of Physical Education, Tianjin Chengjian University, Tianjin, China; 9 Department of Biology, Soonchunhyang University, Asan, Republic of Korea

**Keywords:** adult males, body composition, cross-sectional study, fat mass, physical fitness, skeletal muscle mass, VO_2_max

## Abstract

**Background:**

Body composition is a key determinant of health and physical performance, yet its association with multidimensional physical fitness in adult male populations remains insufficiently understood. This study aimed to investigate the relationships between body composition parameters and physical performance in Chinese adult men.

**Methods:**

A total of 2,362 adult males were recruited from community-based fitness assessment programs in Hengyang, China. Body composition, including body fat percentage, fat mass, fat-free mass, and skeletal muscle mass, was measured using bioelectrical impedance analysis. Physical performance was evaluated through handgrip strength, vertical jump, push-ups, back strength, and estimated VO₂max. Associations were analyzed using Spearman correlation, principal component analysis (PCA), and multiple linear regression models.

**Results:**

Higher body fat percentage was significantly associated with poorer performance in vertical jump, push-ups, and VO₂max (all *p* < 0.001), whereas skeletal muscle mass showed positive associations with strength-related outcomes, particularly handgrip strength (*β* = 0.384, *p* < 0.001). PCA revealed distinct clustering between adiposity-related variables and performance indicators. Regression analyses indicated that skeletal muscle mass was the strongest predictor of strength, while body fat percentage and age were the main predictors of reduced aerobic capacity. BMI showed weaker and less consistent associations with physical fitness compared with direct body composition indicators.

**Conclusion:**

Body composition was associated with physical fitness among adult men. Higher adiposity was related to lower physical performance, whereas greater lean mass was positively associated with strength outcomes. These findings support the value of body composition assessment for community health monitoring, but longitudinal studies are needed before causal conclusions can be drawn.

## 1 Introduction

Body composition refers to the relative proportions of fat mass, muscle mass, bone mineral content, and body water and is widely recognized as an important indicator of health status and functional capacity (1–3). Previous evidence has demonstrated that body composition characteristics are closely associated with health and physical function. Excessive fat accumulation has been associated with increased risks of chronic diseases and impaired physical function (4, 5), whereas greater muscle mass and fat-free mass are generally linked to improved muscular strength, endurance, and metabolic efficiency (6).

Physical fitness is commonly defined as a multidimensional construct encompassing cardiorespiratory endurance, muscular strength and endurance, flexibility, and body composition (7, 8). Adequate levels of physical fitness are strongly associated with disease prevention, healthy aging, and improved quality of life (9). For example, the Physical Fitness Index (PFI) has been used as a comprehensive indicator reflecting cardiovascular endurance, muscular strength, and exercise adaptability (10). Therefore, understanding the relationship between body composition and physical fitness has important implications for health assessment and exercise intervention.

Numerous studies have investigated the relationship between body composition and physical fitness, particularly among adolescents, college students, and older adults (11, 12). Many of these studies have relied on body mass index (BMI) as a surrogate indicator of body composition (13, 14). Large-scale cross-sectional studies have reported that individuals within a normal BMI range generally exhibit better physical fitness, whereas underweight, overweight, and obese individuals tend to show poorer performance in speed-, endurance-, and power-related tests (15, 16). In addition, several studies have reported nonlinear associations between BMI and physical fitness, suggesting that optimal performance may occur within a moderate range rather than at extreme values (17–19). Although BMI remains widely used for its simplicity, it does not distinguish between fat and lean mass and may therefore obscure more precise physiological relationships (20, 21).

Despite these advances, several limitations remain. Most previous studies have focused on specific subgroups, limiting the generalizability of findings to broader adult populations, particularly community-dwelling adult males (22). In addition, relatively few studies have simultaneously incorporated direct body composition measurements and multidimensional physical performance assessments in large samples (23). Consequently, the independent contributions of different body composition components to various domains of physical fitness remain insufficiently understood.

Therefore, the present study aimed to investigate the associations between detailed body composition parameters, including fat mass, fat-free mass, and skeletal muscle mass, and multiple physical performance outcomes in adult males. Specifically, indicators of muscular strength, explosive power, muscular endurance, and cardiorespiratory fitness were evaluated to provide a comprehensive assessment of physical performance. By integrating direct body composition measurements with multidimensional fitness assessments in a large community-based sample, this study seeks to provide a more refined understanding of the role of body composition in functional health and practical implications for exercise intervention.

## 2 Materials and methods

### 2.1 Study design

This study employed a cross-sectional design to examine the associations between body composition and physical performance in adult males. The STROBE recommendations for observational studies guided reporting. All measurements were conducted following standardized protocols by trained professionals to improve data reliability and consistency. This study was conducted in accordance with the Declaration of Helsinki and was approved by the Ethics Committee of Hengyang Normal University (Approval No.: 2026LIIW008).

### 2.2 Participants

A total of 2,400 adult males were recruited from Hengyang City, Hunan Province, China, through community-based physical fitness assessment programs conducted during the study period. Participants were consecutively enrolled from individuals attending community-based physical fitness assessment programs; therefore, the study sample was considered a community-based convenience sample rather than a nationally representative population sample. This recruitment strategy may introduce self-selection bias because individuals who voluntarily attend fitness assessment programs may differ from non-participants in health awareness, lifestyle behaviors, and physical activity habits.

Of the 2,400 recruited participants, 2,362 completed all required assessments and were included in the final analysis, yielding a completion rate of 98.4%. 38 participants were excluded due to incomplete measurements or missing data. As recruitment was conducted in an open community assessment setting, the total number of eligible individuals who declined to participate was not systematically recorded; therefore, a formal participation rate could not be determined. Nevertheless, the large sample size and high completion rate support the dataset’s overall reliability. Participants were classified into three residence-occupation groups according to the community assessment records: urban manual workers, urban non-manual workers, and farmers. This classification was used to partially account for differences in occupational and lifestyle context. However, detailed physical activity information, such as leisure-time exercise, sedentary behavior, weekly training frequency, or objectively measured activity volume, was not collected. Therefore, the occupational group should be interpreted only as a crude contextual variable, not as a direct measure of physical activity level. The present analysis was restricted to adult men because the available community fitness assessment dataset used for this study included male adult participants. In addition, sex-related differences in body composition, fat distribution, skeletal muscle mass, hormonal profiles, and physical performance may influence associations between body composition and fitness.

**Figure 1:**
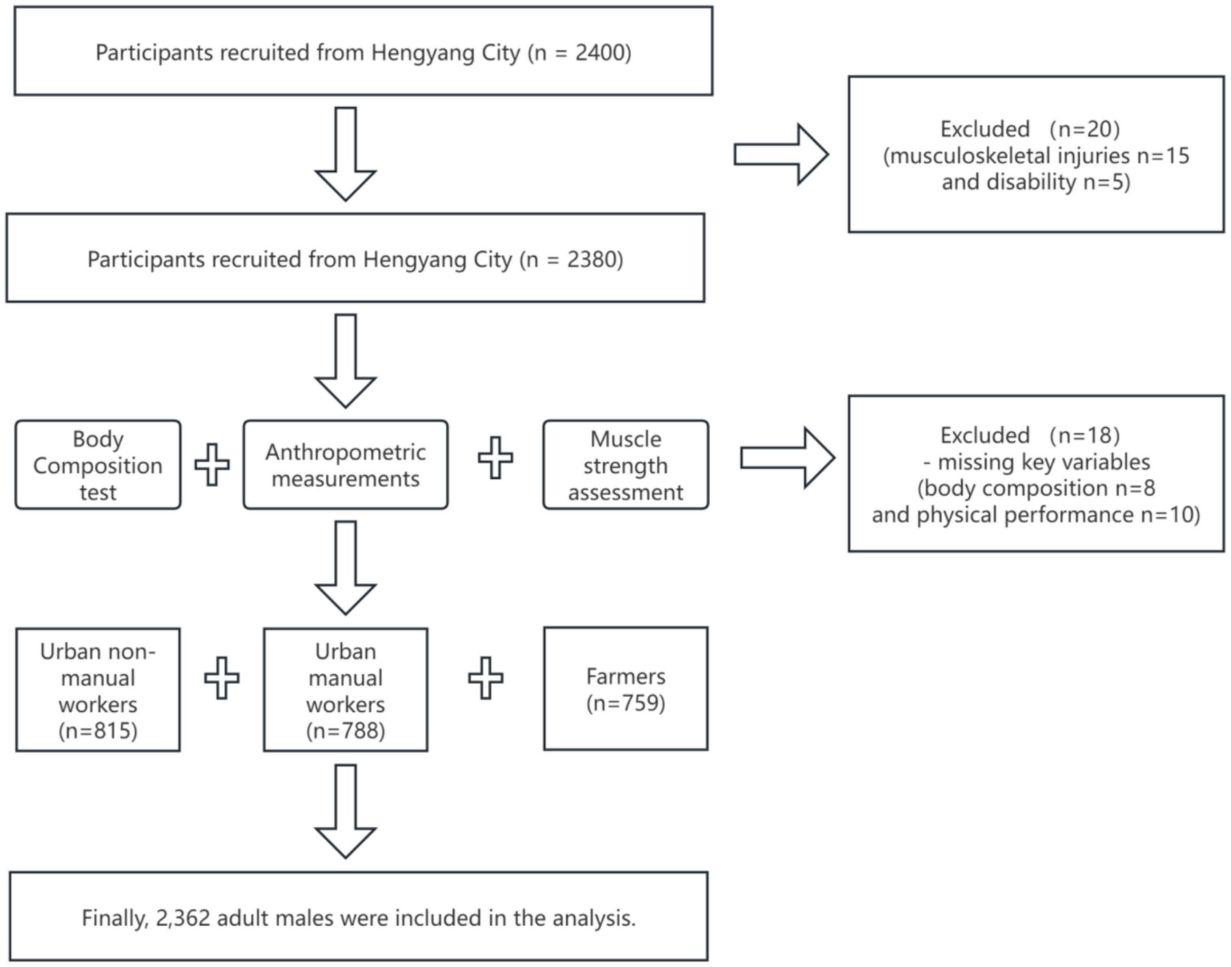
Flowchart of participant recruitment, exclusion, and final inclusion in the study.

Participants were eligible for inclusion if they met the following criteria: (1) male adults aged ≥18 years; (2) no self-reported physician-diagnosed acute illness, unstable cardiovascular or respiratory disease, neurological disorder, severe musculoskeletal injury, or other condition judged by the assessment staff to make maximal or near-maximal physical testing unsafe; and (3) ability to complete all physical fitness tests independently. Disease status was determined using a pre-test health questionnaire and an interview rather than a clinical examination or a medical record review. Exclusion criteria were applied in two stages. First, 20 participants were excluded due to musculoskeletal injuries (*n* = 15) or physical disabilities (*n* = 5). Second, among the remaining participants, 18 were excluded for missing key variables, including body composition (*n* = 8) and physical performance data (*n* = 10). Following these procedures, 2,362 participants were included in the complete-case analysis (Figure 1). All participants were informed of the study procedures before testing, and written informed consent was obtained.

### 2.3 Anthropometric, body composition, and physical performance assessments

#### 2.3.1 Procedures and quality control

All anthropometric, body composition, and physical performance assessments were conducted in accordance with the National Physical Fitness Measurement Standards (2023 revision) and the operating manuals for the Jianmin GMCS series devices (Xindong Huateng Sports Equipment Co., Ltd., Beijing, China). The Jianmin electronic physical fitness assessment system and related GMCS-series devices have been used in previous field-based physical fitness studies in China, including assessments of body size, body composition, handgrip strength, back strength, push-up performance, vertical jump, and estimated VO₂max (24, 25). In addition, the standardized Chinese physical fitness testing protocol has been reported to have acceptable validity and test–retest reliability in population-based fitness assessment settings (26). Before testing each day, trained assessors checked each device’s operational status, verified zeroing and calibration according to the manufacturer’s instructions, and confirmed that the testing environment and participant preparation met the standardized requirements. Participants received uniform instructions and demonstrations before each test. For measures requiring repeated trials, the same standardized trial number, rest interval, and recording rule were applied across participants. The device software automatically recorded or calculated test values where applicable, and assessors immediately reviewed abnormal or incomplete results. These procedures were used to support the validity, reliability, and reproducibility of measurements obtained with the Jianmin assessment systems.

#### 2.3.2 Anthropometric measurements

Age was recorded based on official identification documents. Body height was measured to the nearest 0.1 cm using a stadiometer (Jianmin GMCS-SGJ3, Xindong Huateng Sports Equipment Co., Ltd., Beijing, China), with participants standing barefoot and upright. Body weight was measured to the nearest 0.1 kg using a digital scale (Jianmin GMCS-RCS3, Xindong Huateng Sports Equipment Co., Ltd., Beijing, China), with participants wearing light clothing and no shoes. Body mass index (BMI) was calculated as weight divided by height squared (kg/m^2^).

#### 2.3.3 Body composition assessment

Body composition was assessed using a bioelectrical impedance analysis (BIA) device (Jianmin GMCS-TZL3, Xindong Huateng Sports Equipment Co., Ltd., Beijing, China). The GMCS-TZL3 is a body composition assessment device used in Chinese national physical fitness monitoring settings and conforms to the requirements of the National Physical Fitness Measurement Standards (2023 revision). Participants were instructed to refrain from vigorous physical activity, food intake, and caffeine consumption for at least 2 h before testing. Measurements were performed with participants standing, in accordance with the manufacturer’s guidelines.

The following variables were obtained automatically from the device software: body fat percentage (BF, %), fat mass (FM, kg), fat-free mass (FFM, kg), skeletal muscle mass (SMM, kg), and trunk muscle mass (TMM, kg). These BIA-derived estimates were generated using manufacturer-embedded prediction algorithms. Because the specific prediction equations are proprietary and were not publicly available to the investigators, this issue was acknowledged as a methodological limitation.

#### 2.3.4 Muscle strength assessment

##### 2.3.4.1 Handgrip strength

Handgrip strength (HGS) was measured using a digital hand dynamometer (Jianmin GMCS-WCS3, Xindong Huateng Sports Equipment Co., Ltd., Beijing, China). Participants performed the test in a standing position with the arm fully extended at the side without touching the body. Two trials were performed for each hand, and the highest value was recorded.

##### 2.3.4.2 Back strength

Back strength (BS) was assessed using a back dynamometer (Jianmin GMCS-BLJ3, Xindong Huateng Sports Equipment Co., Ltd., Beijing, China). Participants stood on the platform with knees slightly flexed and pulled the handle upward with maximal effort while maintaining an upright posture. Two trials were conducted, and the highest value was used for analysis. Back dynamometry has shown good reliability for assessing maximal isometric trunk extensor strength in healthy populations (27).

##### 2.3.4.3 Muscular endurance assessment

Upper-body muscular endurance was assessed using a 60-s push-up test. Participants were instructed to perform as many standard push-ups as possible within 60 s while maintaining proper technique. The starting position required the body to be aligned in a straight line, with hands placed shoulder-width apart and arms fully extended. During each repetition, participants lowered their bodies until their elbows reached approximately 90 degrees, then extended their arms to return to the starting position. Only correctly executed repetitions were counted.

The total number of valid repetitions completed within the allotted time was recorded as the final score. The test was conducted using a standardized push-up testing system (Jianmin GMCS-FWC3, Xindong Huateng Sports Equipment Co., Ltd., Beijing, China), which ensured consistency in timing, data recording, and performance monitoring. The push-up test is widely used for assessing upper-body muscular endurance and has demonstrated acceptable reliability and criterion validity in young adults (28).

##### 2.3.4.4 Lower-body power assessment

Vertical jump (VJ) performance was assessed using a jump measurement system (Jianmin GMCS-GTT3, Xindong Huateng Sports Equipment Co., Ltd., Beijing, China). Participants performed a maximal countermovement jump with hands placed on the hips to minimize arm swing. Two trials were conducted, and the best performance was recorded.

##### 2.3.4.5 Cardiorespiratory fitness assessment

Maximal oxygen uptake (VO₂max) was estimated using the Jianmin GMCS-GLC3 cardiopulmonary endurance testing system based on the cycle-ergometer method (Xindong Huateng Sports Equipment Co., Ltd., Beijing, China). The GMCS-GLC3 system is an electronically controlled cycle-ergometer system used for national physical fitness assessment in China and conforms to the National Physical Fitness Measurement Standards (2023 revision). The device uses a non-contact electromagnetic resistance system with a power range of 1–1,000 W, 1-W increments, and an accuracy of ±1 W. Heart rate was continuously monitored using an optical upper-arm heart-rate sensor with a measurement range of 0–300 beats·min^−1^ and an accuracy of ±1 beat·min^−1^. Before testing, participants completed a standardized warm-up and were instructed to avoid strenuous exercise, alcohol, and caffeine intake. During testing, participants performed an incremental cycling protocol while maintaining a constant pedaling cadence of 50 rpm. According to the standardized protocol embedded in the system, workload was increased progressively in stages. For male participants, the initial workload was set at 100 W and increased sequentially to 175 W and 250 W, with each stage lasting 3 min. Throughout the test, the system displayed real-time heart rate, workload, and cadence for continuous monitoring. The test was terminated if participants were unable to maintain the required cadence despite verbal encouragement, voluntarily requested to stop, or exhibited abnormal symptoms, including chest pain, dizziness, unusual dyspnea, or abnormal heart-rate responses. Following completion of the protocol, estimated maximal oxygen uptake was automatically calculated using the manufacturer-embedded algorithm, based on workload and heart rate responses, and expressed relative to body weight (mL·kg^−1^·min^−1^).

### 2.4 Statistical analysis

All statistical analyses were performed using R software (version 4.0.0; R Foundation for Statistical Computing, Vienna, Austria) and GraphPad Prism (version 10.4.2; GraphPad Software, USA).

Continuous variables are presented as mean ± standard deviation (SD), while categorical variables are expressed as frequencies and percentages. The normality of data distribution was assessed using the Shapiro–Wilk test. Homogeneity of variances was evaluated using Levene’s test. For group comparisons, one-way analysis of variance (ANOVA) was applied for normally distributed variables, followed by Bonferroni *post hoc* tests for multiple pairwise comparisons. For the three occupational groups, the Bonferroni-adjusted threshold for pairwise post hoc comparisons was 0.05/3 = 0.0167. Non-normally distributed variables were analyzed using appropriate nonparametric methods where necessary. Categorical variables were compared using the chi-square test.

Before formal statistical analyses, pairwise relationships among variables were visually explored using a scatterplot matrix to assess the direction, strength, and potential nonlinearity of associations, and to detect outliers. Correlations between body composition variables and physical performance outcomes were examined using Spearman’s rank correlation coefficients.

Multiple linear regression analyses were conducted to identify independent predictors of key physical performance outcomes, including handgrip strength, vertical jump height, and maximal oxygen uptake (VO₂max). Each outcome variable was modeled separately. To examine the potential influence of expressing VO₂max relative to body mass, absolute VO₂max (L·min^−1^) was calculated as relative VO₂max × body weight / 1,000 and used in a sensitivity analysis. Independent variables included body fat percentage, skeletal muscle mass, age, body mass index (BMI), and occupational group. Covariates were selected based on prior literature, biological plausibility, and availability in the dataset. Important potential confounders, including leisure-time physical activity, occupational physical activity intensity, dietary intake, smoking, alcohol consumption, socioeconomic status, medication use, and comorbidities, were not available and therefore could not be included in the primary models. The occupational group was treated as a categorical variable and entered into the models as a dummy variable, with farmers as the reference category. Before model fitting, multicollinearity among independent variables was assessed using variance inflation factors (VIF). A VIF value >5 was considered indicative of potential multicollinearity, and values >10 indicated serious multicollinearity (29). Model assumptions were evaluated using residual plots, tests of residual normality, tests of homoscedasticity, and tests of independence of errors (Durbin–Watson test).

Before principal component analysis (PCA), all continuous variables were standardized using z-scores to eliminate scale effects and ensure comparability. PCA was performed on the correlation matrix as an exploratory dimension-reduction and visualization analysis to describe clustering among body composition and physical performance variables, rather than as a primary hypothesis test. PCA was conducted using the “stats” and “FactoMineR” packages in R, and the results were visualized with “factoextra.” Sampling adequacy was evaluated using the Kaiser-Meyer-Olkin (KMO) statistic, and the suitability of the correlation matrix for PCA was assessed using Bartlett’s test of sphericity. Components were interpreted using eigenvalues, explained variance, scree-plot inspection, and loading patterns. For correlation analyses involving 15 body composition-performance tests, the Bonferroni-adjusted significance threshold was 0.05/15 = 0.0033; All statistical tests were two-sided.

Missing data were handled using complete-case analysis because only 18 of 2,400 screened participants (0.75%) had missing key body composition or physical performance variables. The number and reasons for exclusions are shown in Figure 1. No imputation was performed.

## 3 Results

### 3.1 Results of participant characteristics

Participant characteristics across occupational groups are presented in Table 1. No significant differences were observed in age (*p* = 0.259) or body weight (*p* = 0.904), indicating that the groups were comparable at baseline.

**Table 1:** Participant characteristics, body composition, and physical performance across occupational groups.

General characteristics	Overall (*n* = 2,362)	Urban non-manual workers (*n* = 815)	Urban manual workers (*n* = 788)	Farmers (*n* = 759)	*p* for group difference
Age (year)	38.47 ± 11.84	38.01 ± 11.89	38.43 ± 11.93	38.99 ± 11.69	0.259
Height (cm)	170.22 ± 5.79	170.43 ± 5.57	170.56 ± 5.97	169.66 ± 5.79	0.004
Weight (kg)	71.00 ± 10.17	70.87 ± 9.82	71.06 ± 9.84	71.08 ± 10.87	0.904
BMI	24.5 ± 3.3	24.4 ± 3.1	24.4 ± 3.0	24.7 ± 3.4	0.03
Body fat percentage (%)	22.62 ± 5.76	22.52 ± 5.62	22.28 ± 5.61	23.10 ± 6.04	0.016
Handgrip strength (kg)	41.89 ± 7.36	42.49 ± 7.12	42.16 ± 7.68	40.95 ± 7.17	<0.001
Vertical jump (cm)	37.63 ± 11.00	38.22 ± 10.44	38.59 ± 11.63	36.01 ± 10.74	<0.001
Push-ups (reps)	28.89 ± 15.80	30.61 ± 16.22	30.64 ± 16.70	25.23 ± 13.61	<0.001
Back strength (kg)	120.32 ± 24.04	123.32 ± 24.03	119.69 ± 23.64	117.74 ± 24.13	<0.001
VO2max (mL·kg^−1^·min^−1^)	43.80 ± 15.29	44.00 ± 15.37	44.66 ± 16.02	42.70 ± 14.36	0.038
Fat-free mass (kg)	54.38 ± 5.48	54.45 ± 5.12	54.78 ± 5.26	53.88 ± 6.02	0.005
Fat mass (kg)	16.54 ± 6.15	16.41 ± 5.92	16.28 ± 5.88	16.95 ± 6.64	0.077
Skeletal muscle mass (kg)	50.99 ± 5.22	51.07 ± 4.80	51.39 ± 4.93	50.50 ± 5.87	0.003
Trunk muscle mass (kg)	27.11 ± 2.77	27.17 ± 2.57	27.35 ± 2.65	26.80 ± 3.07	<0.001

Values are mean ± SD. *p* values are from one-way ANOVA across the three occupational groups.

Significant differences were identified in several anthropometric and body composition variables. Farmers had a lower mean height compared with urban manual and non-manual workers (*p* = 0.004). In addition, body mass index (BMI) differed significantly among groups (*p* = 0.030), with farmers exhibiting slightly higher BMI values compared with urban groups. Body fat percentage was also significantly higher in farmers than in urban groups (*p* = 0.016). In contrast, urban non-manual workers exhibited higher fat-free mass, skeletal muscle mass, and trunk muscle mass compared with farmers (all *p* < 0.01).

Physical performance outcomes also differed significantly across occupational groups. Urban manual workers demonstrated the highest values for handgrip strength, vertical jump, push-up performance, and back strength, with all comparisons statistically significant (all *p* < 0.001). Urban non-manual workers showed performance levels comparable to those of urban manual workers, though slightly lower on several strength-related measures. In contrast, farmers consistently exhibited lower performance across these variables. For cardiorespiratory fitness, VO₂max differed significantly among groups (*p* = 0.038), with urban non-manual workers showing higher values than farmers.

### 3.2 Results of PCA

The data were suitable for PCA, with an overall KMO value of 0.726 and a significant Bartlett’s test of sphericity (χ^2^ = 33016.463, df = 45, *p* < 0.001). The first two principal components explained 65.2% of the total variance, with PC1 accounting for 40.1% and PC2 accounting for 25.1%. Because PCA was used for exploratory descriptive analysis, the extracted components were interpreted cautiously and not treated as evidence of causal pathways or stable latent constructs.

In the loading matrix, PC1 was mainly characterized by high absolute loadings for fat-free mass (FFM; −0.948), skeletal muscle mass (SMM; −0.946), trunk muscle mass (TMM; −0.932), fat mass (FM; −0.762), and body fat percentage (BF%; −0.593) (Table 2). This pattern suggests that PC1 primarily represents variation in body composition, particularly lean-mass-related variables, while acknowledging that the direction of PCA loadings is arbitrary.

**Table 2:** Principal component analysis loading matrix for body composition and physical performance variables.

Variable	PC1	PC2	PC3
BF%	−0.593	−0.618	−0.140
HGS	−0.464	0.513	−0.388
VJ	−0.089	0.688	−0.054
PU	−0.012	0.758	0.021
BS	−0.401	0.610	−0.376
VO₂max	0.186	0.382	0.701
FFM	−0.948	0.086	0.210
FM	−0.762	−0.517	−0.064
SMM	−0.946	0.088	0.216
TMM	−0.932	0.123	0.209

PC2 showed relatively high loadings for push-ups (PU; 0.758), vertical jump (VJ; 0.688), back strength (BS; 0.610), handgrip strength (HGS; 0.513), body fat percentage (BF%; −0.618), and fat mass (FM; −0.517). This pattern suggests an exploratory contrast between performance-related variables and adiposity-related variables. VO₂max had its highest loading on PC3 (0.701), suggesting that estimated aerobic capacity contributed less strongly to the first two components than the strength- and body-composition-related variables.

**Figure 2:**
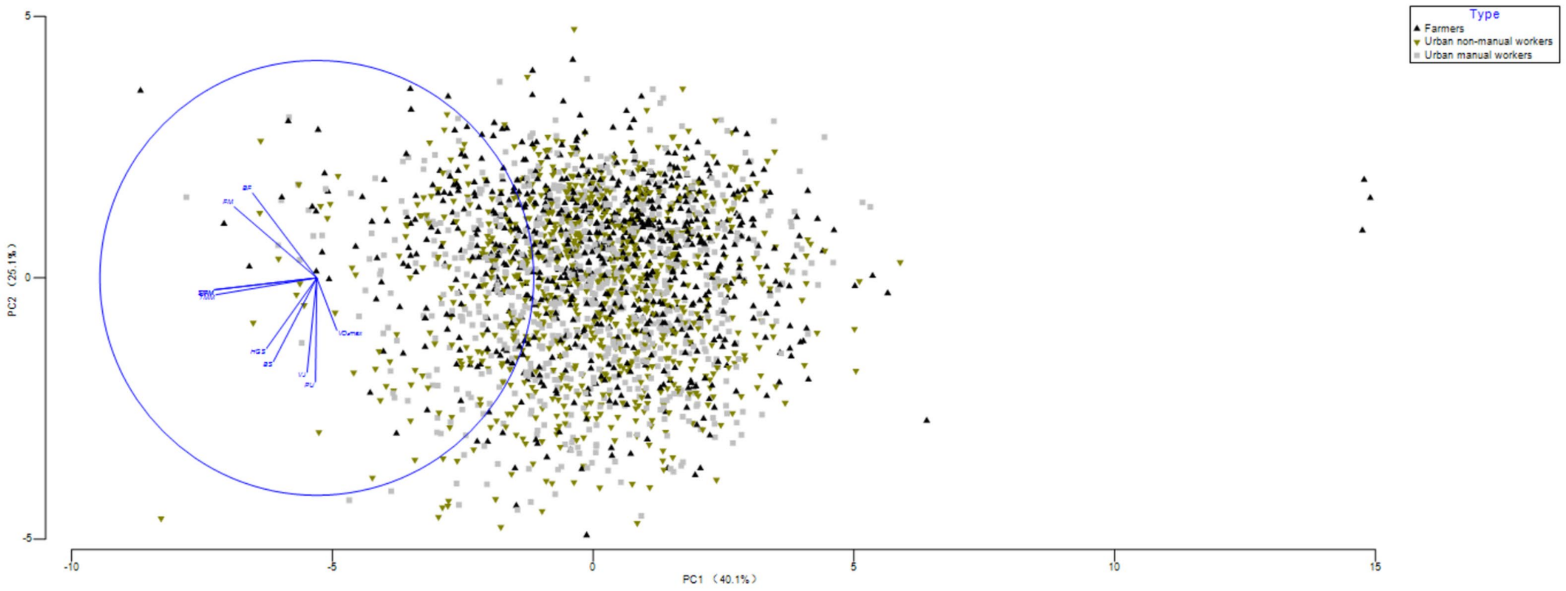
PCA biplot of body composition and physical performance variables across occupational groups.

The PCA biplot (Figure 2) showed that most individuals clustered near the center, with no clear separation among occupational groups. Therefore, the PCA results were interpreted only as a descriptive visualization of multivariate clustering. The full loading matrix is provided in Supplementary Table S1 to improve transparency.

### 3.3 Results of correlation analysis

**Figure 3:**
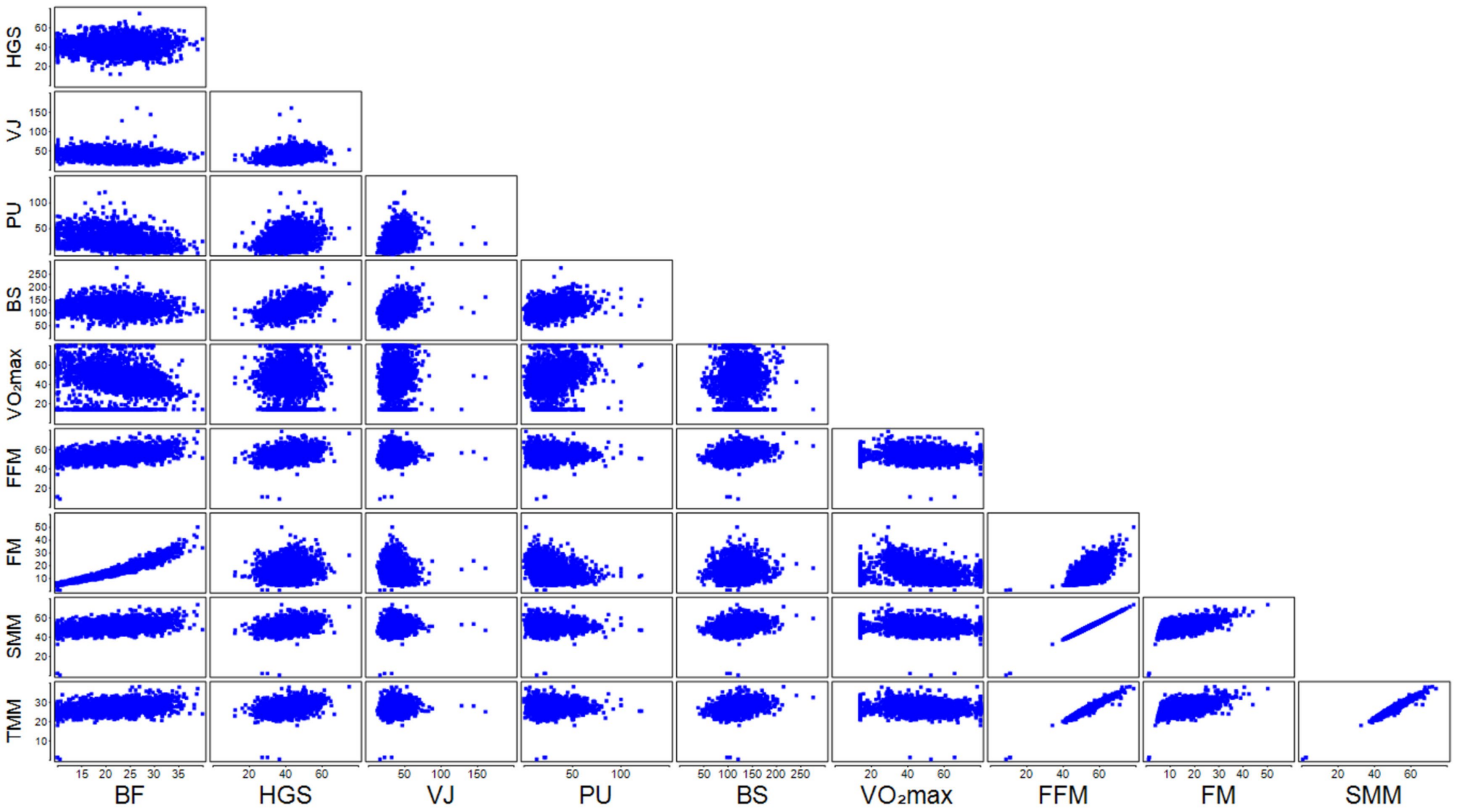
Pairwise scatterplot matrix showing relationships among body composition, physical performance, and aerobic capacity variables.

Visual inspection of the scatterplot matrix revealed clear patterns of association among variables. Lean mass-related indicators (FFM, SMM, and TMM) showed strong positive linear relationships, suggesting substantial overlap among these measures. In contrast, fat-related variables (BF and FM) were positively associated with each other but were generally inversely associated with lean mass indicators. Among performance variables, handgrip strength demonstrated a moderate positive association with muscle-related indices, whereas VO₂max exhibited a negative trend with body fat percentage. Other performance measures (VJ, PU, and BS) displayed more heterogeneous relationships with body composition variables, with dispersion patterns indicating potential skewness and outliers in certain variables (Figure 3).

Consistent with these visual patterns, Spearman correlation analysis (Table 3) further quantified the associations between body composition and physical performance outcomes. Body fat percentage showed inverse correlations with vertical jump, push-up performance, and relative VO₂max that remained significant after Bonferroni correction (all *p* < 0.001). However, the magnitude of these correlations varied from weak to moderate, indicating that statistical significance should not be interpreted as strong practical relevance. The correlation between body fat percentage and back strength was statistically weak and did not meet the Bonferroni-adjusted significance threshold (r = −0.046, 95% CI: −0.086 to −0.006; *p* = 0.026). Body fat percentage was not meaningfully associated with handgrip strength (r = 0.035, 95% CI: −0.005 to 0.075; *p* = 0.088).

**Table 3:** Spearman correlation coefficients between body composition variables and physical performance outcomes.

Predictor	Handgrip strength (kg)	Vertical jump (cm)	Push-ups (reps)	Back strength (kg)	VO2max (mL·kg^−1^·min^−1^)
Body fat percentage (%)	0.035 (*p* = 0.088; −0.011 to 0.070)	−0.261 (*p* = <0.001; −0.328 to −0.254)	−0.346 (*p* = <0.001; 0.389 to −0.318)	−0.046 (*p* = 0.026; −0.097 to −0.017)	−0.250 (*p* = <0.001; −0.355 to −0.283)
Skeletal muscle mass (kg)	0.360 (*p* = <0.001; 0.336 to 0.405)	0.115 (*p* = <0.001; 0.092 to 0.171)	0.056 (*p* = 0.006; 0.036 to 0.116)	0.318 (*p* = <0.001;0.293 to 0.365)	−0.099 (*p* = <0.001;−0.154 to −0.074)
Trunk muscle mass (kg)	0.380 (*p* = <0.001; 0.358 to 0.427)	0.119 (*p* = <0.001; 0.092 to 0.171)	0.085 (*p* = <0.001; 0.059 to 0.138)	0.341 (*p* = <0.001; 0.314 to 0.385)	−0.087 (*p* = <0.001; −0.142 to −0.062)

Values are correlation coefficients with 95% confidence intervals. The Bonferroni-adjusted significance threshold was *p* < 0.0033. Associations with *p* < 0.05 but ≥0.0033 were considered nominally significant only.

Muscle-related variables were positively associated with strength-related outcomes, particularly handgrip strength and back strength. Skeletal muscle mass showed moderate correlations with handgrip strength (r = 0.360, 95% CI: 0.325 to 0.395; *p* < 0.001) and back strength (r = 0.318, 95% CI: 0.281 to 0.354; *p* < 0.001). However, some statistically significant associations were trivial or very weak. For example, the association between skeletal muscle mass and push-up performance was trivial in magnitude (r = 0.056, 95% CI: 0.016 to 0.096; *p* = 0.006) and did not meet the Bonferroni-adjusted significance threshold of *p* < 0.0033. Skeletal muscle mass and trunk muscle mass were negatively correlated with relative VO₂max. Still, the effect sizes were very weak, possibly reflecting the influence of expressing aerobic capacity relative to body mass. The Bonferroni-adjusted significance threshold for the correlation analyses was *p* < 0.0033. Therefore, only associations with *p* values below this threshold were interpreted as statistically significant after correction for multiple comparisons. Associations with *p* values below 0.05 but above 0.0033, such as BF% with back strength (r = −0.046, *p* = 0.026) and SMM with push-up performance (r = 0.056, *p* = 0.006), were considered nominally significant only and were not interpreted as robust associations.

### 3.4 Results of multiple linear regression analyses

Multiple linear regression analyses were conducted to identify independent predictors of key physical performance outcomes (Table 4). Multicollinearity diagnostics showed that VIF values were generally within an acceptable range. Across the fully adjusted models, VIF values were 1.23 for age, 4.25 for BMI, 3.85 for BF%, 2.05 for SMM, 1.37 for urban manual workers, and 1.38 for urban non-manual workers. BMI had the highest VIF, indicating moderate collinearity with body composition variables, but no predictor exceeded the 10 threshold for serious multicollinearity. Therefore, all prespecified predictors were retained.

**Table 4:** Multiple linear regression models for physical performance outcomes.

Outcome	Predictor	B	SE	Standardized *β*	95% CI	*p* value	Adjusted R^2^
Handgrip strength (kg)	Body fat percentage (%)	0.013	0.047	0.01	−0.080 to 0.105	0.790	0.161
	Skeletal muscle mass (kg)	0.541	0.038	0.384	0.467 to 0.616	<0.001	
	Age (years)	−0.084	0.013	−0.135	−0.109 to −0.058	<0.001	
	BMI (kg/m^2^)	−0.229	0.099	−0.1	−0.423 to −0.034	0.021	
	Urban manual workers	1.093	0.341	0.071	0.424 to 1.763	0.001	
	Urban non-manual workers	0.633	0.345	0.041	−0.044 to 1.310	0.067	
Vertical jump (cm)	Body fat percentage (%)	−0.131	0.064	−0.069	−0.257 to −0.005	0.042	0.304
	Skeletal muscle mass (kg)	0.254	0.052	0.12	0.152 to 0.355	<0.001	
	Age (years)	−0.424	0.018	−0.457	−0.459 to −0.390	<0.001	
	BMI (kg/m^2^)	−0.5	0.135	−0.146	−0.765 to −0.235	<0.001	
	Urban manual workers	1.428	0.465	0.062	0.517 to 2.340	0.002	
	Urban non-manual workers	1.877	0.47	0.08	0.955 to 2.799	<0.001	
VO_2_max	Body fat percentage (%)	−0.529	0.102	−0.199	−0.729 to −0.329	<0.001	0.092
	Skeletal muscle mass (kg)	−0.287	0.082	−0.098	−0.448 to −0.125	<0.001	
	Age (years)	−0.249	0.028	−0.193	−0.304 to −0.194	<0.001	
	BMI (kg/m^2^)	0.206	0.214	0.043	−0.215 to 0.626	0.338	
	Urban manual workers	0.967	0.738	0.03	−0.480 to 2.414	0.190	
	Urban non-manual workers	1.691	0.747	0.052	0.226 to 3.156	0.024	

For handgrip strength, skeletal muscle mass was the strongest positive predictor (*β* = 0.384, *p* < 0.001), whereas age (*β* = −0.135, *p* < 0.001) and BMI (*β* = −0.100, *p* = 0.021) were negatively associated with performance. Occupational status also had a significant effect, with urban manual workers showing higher handgrip strength than the reference group (*p* = 0.001), while the association for urban non-manual workers did not reach statistical significance (*p* = 0.067). Body fat percentage was not a significant predictor (*p* = 0.790). The model explained 16.1% of the variance.

For vertical jump performance, age was the most influential predictor (*β* = −0.457, *p* < 0.001), followed by BMI (β = −0.146, *p* < 0.001), both of which were negatively associated with performance. Skeletal muscle mass was positively associated with vertical jump (β = 0.120, *p* < 0.001), whereas body fat percentage showed only a weak borderline negative association (β = −0.069, *p* = 0.042). Because this result would not remain significant after a conservative correction for multiple comparisons, it should be interpreted with caution. Both urban manual and non-manual workers exhibited significantly higher vertical jump performance compared with the reference group (both *p* ≤ 0.002). This model accounted for 30.4% of the variance.

For relative VO₂max, body fat percentage was the strongest predictor (*β* = −0.199, *p* < 0.001), indicating that higher adiposity was associated with lower relative cardiorespiratory fitness. Age (β = −0.193, *p* < 0.001) and skeletal muscle mass (β = −0.098, *p* < 0.001) were also negatively associated with relative VO₂max. Occupational status showed a limited effect, with only urban non-manual workers demonstrating a modest positive association (*p* = 0.024). BMI was not significantly associated with relative VO₂max (*p* = 0.338). However, the model explained only 9.2% of the variance, indicating that the available predictors did not capture a substantial proportion of the variance in individual differences in relative VO₂max. Unmeasured variables, such as physical activity level, training history, smoking, diet, comorbidities, and measurement-related factors, may have contributed to the remaining variance. Therefore, the VO₂max regression findings should be interpreted with caution and not considered sufficient for individual-level prediction. In the sensitivity analysis using absolute VO₂max (L·min^−1^), skeletal muscle mass showed a positive association with absolute VO₂max (standardized β = 0.135, *p* < 0.001). In contrast, it was negatively associated with relative VO₂max in the primary model (standardized β = −0.098, *p* < 0.001). This change in direction suggests that the negative association between SMM and relative VO₂max may partly reflect the influence of expressing aerobic capacity per kilogram of body mass.

Overall, skeletal muscle mass was most consistently associated with strength-related performance, whereas adiposity and age were associated with relative VO₂max. However, the modest explanatory power of the VO₂max model suggests that body composition variables alone provide only a limited explanation of aerobic capacity in this sample.

## 4 Discussion

### 4.1 Main findings and interpretation

This study suggests that body composition indicators are associated with several domains of physical fitness in adult men, but the strength of these associations varied by outcome. Higher adiposity was related to lower relative VO₂max, push-up performance, and vertical jump performance, whereas skeletal muscle mass was most consistently associated with strength-related outcomes. However, several statistically significant correlations were small, and the regression model for relative VO₂max explained only a modest proportion of the variance. These findings therefore support cautious interpretation rather than broad prediction of individual fitness from body composition alone.

The contrasting associations of fat mass and lean mass with physical performance are consistent with general biomechanical and physiological considerations. Excess adiposity may increase the mechanical load during movement and elevate the energetic cost of weight-bearing activities, potentially reducing efficiency in endurance- and power-related tasks (30). In contrast, fat-free mass, particularly skeletal muscle mass, plays an important role in force production and neuromuscular function. Greater muscle mass may contribute to improved strength and power output and may also support metabolic regulation, including glucose uptake and mitochondrial function (31, 32). These mechanisms may partly explain the positive associations between lean mass and strength-related indicators observed in the present study.

Previous studies have also reported that the relationship between BMI and physical fitness may be nonlinear, with some evidence of an inverted-U association, in which optimal performance is often observed within the normal BMI range (33). This suggests that both insufficient and excessive body mass could negatively affect physical function. However, because BMI does not distinguish between fat mass and lean mass, individuals with similar BMI values may still differ substantially in body composition. This limitation may partly account for the relatively inconsistent associations involving BMI observed in the present study. Therefore, body composition indicators may provide more physiologically relevant information regarding physical performance than BMI alone.

### 4.2 Comparison with previous studies and practical implications

The findings of the present study are generally consistent with previous research showing that higher fat mass is associated with lower physical fitness. In contrast, lean mass is typically positively associated with performance. For example, a cross-sectional study of Chinese medical students reported that fat mass was the only body composition variable significantly associated with lower physical fitness scores, particularly among male participants (22). Similarly, studies in adult populations have suggested that muscle strength is positively associated with fat-free mass, whereas cardiorespiratory fitness is negatively associated with adiposity (34). These findings are broadly consistent with the present results and suggest that the associations between body composition and physical fitness may be observed across different populations and age groups. Importantly, the present study extends previous findings by focusing on community-dwelling adult men rather than specific subgroups such as students or athletes. This may improve the broader applicability of the findings. In addition, the use of multiple analytical approaches, including correlation analysis, principal component analysis, and regression modeling, provides complementary evidence supporting the observed associations.

From a practical perspective, these findings suggest that BMI alone may provide an incomplete description of fitness-related body composition. Direct body composition assessment may be useful as an adjunct in community fitness evaluation, particularly for distinguishing adiposity from lean mass. However, because the study was cross-sectional and did not include intervention or longitudinal follow-up data, any implications for training programs or health monitoring should be considered hypothesis-generating rather than prescriptive.

In addition to body composition, occupational group differences were observed for several physical performance outcomes. These differences may reflect variation in daily activity patterns, lifestyle, socioeconomic factors, or health behaviors. Previous studies have suggested that occupational physical activity, sedentary time, leisure-time exercise, and occupational characteristics may be associated with body composition, health indicators, and physical fitness in different ways (35–39). However, the study did not include objective physical activity measurements or a detailed occupational workload assessment. Therefore, occupational explanations, including those related to the physical activity paradox, remain speculative and should be interpreted as contextual possibilities rather than direct findings of this study.

Previous studies have also reported that occupational physical activity and leisure-time exercise may have different associations with health and fitness, and that workplace physical activity interventions may have variable effects depending on occupational context (40–42). Nevertheless, the present data cannot determine whether occupational workload, leisure-time exercise, recovery, or other lifestyle factors explained the group differences observed here.

Taken together, the present results indicate that body composition is associated with several fitness indicators in this community-based sample, but the observed associations should be interpreted in light of the exploratory PCA, modest explanatory power in regression, and the absence of direct physical activity data.

### 4.3 Limitations and future directions

Several limitations of this study should be acknowledged. First, due to the cross-sectional design, causal relationships cannot be established. Although significant associations were observed, the directionality of these relationships remains unclear, and reverse causation cannot be excluded (20). Previous research suggests that body composition and physical fitness may influence each other bidirectionally (43). Second, participants were recruited from community-based physical fitness assessment programs using a convenience sampling approach. Therefore, the sample may not be fully representative of the general adult male population, and individuals who voluntarily participated in fitness assessments may have differed from non-participants in health awareness, lifestyle behaviors, physical activity habits, or underlying health status. This self-selection bias may limit the external validity of the findings. Third, although the occupational group was included in the regression models as a contextual covariate, detailed physical activity variables, such as leisure-time exercise, occupational physical activity intensity, sedentary behavior, weekly exercise frequency, dietary intake, smoking status, alcohol consumption, socioeconomic status, medication use, and comorbidities, were not comprehensively assessed. These factors may influence both body composition and physical fitness and, therefore, could contribute to residual confounding. Fourth, body composition was assessed using bioelectrical impedance analysis. Although the Jianmin GMCS-TZL3 device is used in Chinese national physical fitness monitoring settings and is practical for large-scale field assessment, BIA is less precise than gold-standard methods such as dual-energy X-ray absorptiometry (44). BIA-derived estimates may be affected by hydration status, recent food intake, body geometry, and device-specific prediction algorithms. Because the manufacturer-embedded prediction equations were proprietary and not publicly available, we could not independently verify whether these equations had been specifically validated in Chinese adult males. Therefore, systematic or random estimation error in fat mass, fat-free mass, skeletal muscle mass, and trunk muscle mass cannot be excluded, and the direction and magnitude of such error cannot be determined in the present study. Fifth, VO₂max was estimated using a device-based cycle ergometer protocol rather than being measured directly by respiratory gas analysis. Although the GMCS-GLC3 system conforms to the National Physical Fitness Measurement Standards (2023 revision), the present study reported the standardized workload progression, stage duration, and cadence requirements used during testing. However, the manufacturer-embedded VO₂max estimation equation was proprietary and not publicly available to the investigators. Therefore, estimated VO₂max values should be interpreted as device-derived estimates rather than directly measured maximal oxygen uptake, and comparability with studies using respiratory gas analysis may be limited. This may limit reproducibility and comparability with studies using directly measured VO₂max. The relatively high variability in estimated VO₂max (43.80 ± 15.29 mL·kg^−1^·min^−1^) may reflect the heterogeneity of the community-based sample, including a broad age range, occupational differences, and unmeasured variation in physical activity level, training history, smoking status, and health conditions. Therefore, the VO₂max findings should be interpreted as an estimate of aerobic fitness rather than as a direct measure of maximal oxygen uptake.

Finally, the study population consisted exclusively of adult men, which may limit the generalizability of the findings to women, older adults, or other demographic groups. Because sex-related differences in body composition, fat distribution, skeletal muscle mass, hormonal profiles, and physical performance may influence body composition-fitness associations, future studies should include female participants and perform sex-specific analyses. Future prospective cohort studies and intervention trials are needed to clarify the causal pathways underlying the observed associations. In particular, studies incorporating direct physical activity assessment, comprehensive lifestyle and clinical covariates, repeated body composition measurements, and more precise body composition methods would help strengthen the evidence base.

## 5 Conclusion

This cross-sectional study showed that body composition was associated with physical performance in Chinese adult men. Higher adiposity was related to poorer relative cardiorespiratory fitness, muscular endurance, and lower-body power, whereas greater skeletal muscle mass was positively associated with strength-related outcomes. Compared with BMI, direct body composition indicators may provide more specific information for interpreting functional fitness in community assessments. However, the findings should be interpreted cautiously because the study was cross-sectional, used a convenience sample, lacked key lifestyle covariates, and relied on BIA-derived body composition estimates. Intervention-related implications should therefore be considered hypothetical and require confirmation in longitudinal or experimental studies.

## Data Availability

The raw data supporting the conclusions of this article will be made available by the authors, without undue reservation.
